# Adsorption Application of Choline Chloride Modified MIL-101 (Cr) in Carbon Capture and Storage

**DOI:** 10.3390/ma18102370

**Published:** 2025-05-20

**Authors:** Entian Li, Zuquan Zhang, Minghe Zhou, Pei Yao

**Affiliations:** 1 School of Petroleum Engineering, Changzhou University, Changzhou 213164, China; 13762804663@163.com (Z.Z.); 18661190663@163.com (M.Z.); 2 Department of Chemistry and Materials Engineering, Changzhou Vocational Institute of Engineering, Changzhou 213164, China; yaopeitel@163.com

**Keywords:** carbon capture, metal-organic framework, MIL-101(Cr), choline chloride, CO_2_ adsorption

## Abstract

This study developed a new way of designing choline chloride-modified MOF-based materials with advanced gas adsorption properties. To design better carbon capture materials, MIL-101 (Cr) was prepared using the hydrothermal method, and then was modified with different concentrations of choline chloride in a one-step method to enhance its CO_2_ adsorption capacity. The characterization and experimental results indicated that the modified ChCl-MIL-101(Cr) significantly enhanced the adsorption capacity for CO_2_. Specifically, the 0.075-ChCl-MIL-101(Cr) showed a 61.191% increase in adsorption capacity compared to that of the raw material. Moreover, the regenerated adsorption loss rate of the modified material was below 4%, proving the permanence of the material synthesis. Simulating isotherms using Langmuir and Freundlich equations revealed the non-uniformity of surface bonding.

## 1. Introduction

With the process of industrialization, the emission of greenhouse gases, primarily carbon dioxide (CO_2_), has caused global warming and seriously threatened the development of human society [1]. Moreover, the decrease in pH caused by carbon dioxide and the hazards brought about by carbon dioxide mineralization are also increasing rapidly [2,3]. CO_2_ capture technologies constitute an important approach to solving the above problems [4]. There are four application scenarios for CO_2_ capture: pre-combustion capture, oxygen-enriched combustion, post-combustion capture, and direct air capture. The corresponding CO_2_ concentrations for these scenarios are 15% to 60%, 75% to 90%, 4% to 15%, and 0.02% to 0.04%,respectively [5]. Currently, the absorption method is the most widely used decarbonization technology for high-concentration CO_2_ capture scenarios, with inorganic alkali or organic amine solution as the core material [6]. However, this technology requires high regeneration energy consumption and encounters prominent problems such as pipeline corrosion, resulting in high decarbonization costs [7,8,9]. In recent years, the adsorption method has been widely favored by virtue of its high overall stability and low regeneration energy consumption, especially in the field of low concentration (≤15%) CO_2_ capture, and it is considered part of a new generation of CO_2_ capture technology with great application prospects. The core of the adsorption method lies in developing adsorption materials with high adsorption capacity, high adsorption selectivity, and high stability [10,11,12].

In addition to the implementation of good environmental practices and eco-sustainable energy policies, environmental preservation can be also achieved through the drastic and rapid reduction of global greenhouse gas emissions via their capture. In particular, the effects CO_2_ could be mitigated through the use of the best scientific knowledge available in the development of effective technologies in carbon capture and storage (CCS) or utilization (CCU) [13,14,15]. At present, there are two main CO_2_ adsorption technologies: one is the chemical adsorption method, and the other is the physical adsorption method. The physical absorption method separates CO_2_ by taking advantage of the difference in the solubility of CO_2_ in the aqueous phase. The chemical absorption principle realizes the adsorption of CO_2_ through the acid–base neutralization of CO_2_. Commonly used adsorption materials currently include carbon based materials [16], zeolites [17], silica [18], MOFs [19], as well as other materials for porous adsorption. Among them, MOFs have high crystallinity, a high specific surface area, and adjustable pore structure, demonstrating considerable potential in the field of CO_2_ capture. MOFs possess advantages that other materials cannot achieve, such as a high surface area, the capability to physically adsorb hydrogen, and the separation and storage of CH_4_ and CO_2_ [20]. The MILs in MOFs have excellent stability, and each metal ion has a capped ligand. Employing highly coordinated amino groups for substitution, specific structures and even specific functions can be achieved through the design of raw material molecules [21,22].

Based on various factors such as the interaction force and mechanism between the material and the target pollutant [23], the structure of the material can be modified to synthesize MIL metal frameworks with suspended amino groups, showing stronger CO_2_ adsorption capacity, and can significantly improve the selective adsorption and capture capability of CO_2_. Zhang [24] synthesized MIL-101 (Cr) by using chromium nitrate (Cr(NO_3_)_3_·9(H_2_O)), terephthalic acid, and hydrofluoric acid. Its specific surface area reached 2846.69 m^2^·g^−1^, its pore volume was 2.45 cm^3^·g^−1^, and average pore size was 0.98 nm. Compared with other MIL-101 materials, its crystal structure and adsorption effect were the best. Additionally, Liang Fangfang found that it can adsorb CO_2_ at room temperature and pressure, with improved adsorption capacity and selectivity. MIL-101 (Cr) modified by ED can effectively adsorb greenhouse gas CO_2_ under mild conditions at room temperature and pressure. The modified material MIL-101-ED-0.18 has an adsorption capacity of 2.43 mmol·g^−1^ for CO_2_ at room temperature and pressure, an increase of 14.6% compared to that of before modification, which is higher than other similar materials. The modified material also has greatly improved gas adsorption selectivity, with its CO_2_/N_2_ selective separation coefficient increasing from 11 to 17, an increase of 55.6% compared to that of before modification. Chang [25] fabricated a novel bimetallic MIL-101 (Cr, Mg), and compared with loading a single metal material, the synergistic effect of the bimetallic material was shown to improve its CO_2_/N_2_ adsorption selectivity. The adsorption capacity and pore size of other MOF materials are shown in Table 1.

Choline chloride is also widely used in carbon dioxide absorption applications [31]. Its low melting point solvent (ChCl EG-based DES) formed with ethylene glycol (EG) is a new type of ionic liquid, which has the advantages of low price, low viscosity, easy synthesis, and suitability for large-scale production [32]. However, it still cannot escape the disadvantages of low regeneration energy consumption and high corrosiveness, and ethylene glycol does not have alkaline groups, so its capability to absorb carbon dioxide is relatively weak. Giancarlo developed choline amino acid based ionic liquids ([Ch][His]ILs) using choline (quaternary ammonium cation) instead of imidazole cation, which are almost non-toxic and considered green chemicals [33]. Cheng [34] demonstrated the performance of choline amino acids in absorbing carbon dioxide. The results indicate that when the moisture content is 30%, the CO_2_ absorption increases continuously with increasing pressure. Although the absorption of CO_2_ by a single choline amino acid ionic liquid is hindered by viscosity, its aqueous solution has a good absorption effect [35,36].

Porous materials can be used for the separation and adsorption of gases due to their adsorption principle being physical adsorption and their high porosity and huge specific surface area, as well as their low adsorption kinetics and selectivity [37]. They must, therefore, be changed and activated. The use of active functional groups for surface modification is regarded as one of the most common and effective methods [13]. Carbon dioxide is an acidic gas, and based on physical adsorption, the introduction of basic amine groups can be used to incorporate chemical adsorption, which can significantly enhance the material’s adsorption performance for carbon dioxide [38,39].

However, despite the continuous progress in CO_2_ capture technology, there are still some critical issues that remain unresolved. For instance, most existing adsorption materials face challenges such as high cost, low adsorption capacity under practical conditions, and poor long-term stability. Additionally, the balance between the efficiency of carbon capture and the energy consumption during the adsorption-desorption process has not been well-addressed. These problems severely limit the large-scale application of carbon capture technology. Therefore, in the context of these persistent challenges in the field of CO_2_ capture science, this study aims to develop an efficient and cost-effective solution.

The impact of choline chloride concentration on material structure and CO_2_ adsorption performance is thoroughly examined in this study, and the CO_2_ adsorption behavior of composite materials is analyzed using adsorption isotherm equations. The results show that with an increase in choline chloride concentration, the CO_2_ adsorption trend displayed a volcano pattern, and the CO_2_ adsorption of 0.075-ChCl-MIL-101 (Cr) was the best. MIL-101 (Cr) has a unique pore cage structure that makes it capable of efficiently adsorbing CO_2_. A multilayer porous MOF is created when the composite material and choline chloride mix through coordination and hydrogen bonding. Both the chemical and physical characteristics of the materials are monitored using a variety of characterization techniques. The composite material’s capacity for adsorbing carbon dioxide is determined, and a comprehensive examination of the adsorption process mechanism is conducted.

## 2. Materials and Methods

### 2.1. Materials and Equipment

The materials and equipment are shown in Table 2 and Table 3.

### 2.2. Material Preparation

#### 2.2.1. Preparation of MIL-101 (Cr)

A hydrothermal synthesis method was employed, where 2.4 g of Cr(NO_3_)_3_·9H_2_O was dissolved in 28.6 mL of deionized water. Then, 0.996 g of H_2_BDC was added after thorough stirring, and 0.26 mL of 40% HF was dripped in while stirring. The mixture was then subjected to ultrasonic agitation for 30 min before being transferred to a reaction vessel. The reaction was carried out at 220 °C for 8 h. After cooling, the green product was obtained and subjected to solid-liquid separation using a centrifuge. It was then washed repeatedly with DMF and anhydrous ethanol until the liquid’s color no longer changed. After filtration, the product was placed in an 80 °C oven for drying for 8 h. Finally, the green product was stored in a sealed bag and labeled as MIL-101 (Cr) [40].

#### 2.2.2. Preparation of n-ChCl-MIL-101 (Cr)

N-ChCl-MIL-101 (Cr) was modified using a one-step method. Firstly, 0.996 g of H_2_BDC was dissolved in water, and different concentrations of ChCl were added dropwise for the reaction. Then, 2.4 g of Cr(NO_3_)_3_·9H_2_O was added, and the mixture was shaken in an ultrasonic oscillator for half an hour. Meanwhile, 0.26 mL of 40% concentration HF was added dropwise while stirring. The mixture was then added to a reaction vessel and reacted at 220 °C for 8 h. After cooling, the green product was obtained and subjected to solid–liquid separation using a centrifuge. Then, DMF and anhydrous ethanol were washed back and forth until the liquid color did not change anymore. After filtration, the product was dried in an 80 °C oven for 8 h. Finally, the green product was placed in a sealed bag and labeled with n-ChCl-MIL-101 (Cr) for storage (where n represents the concentration of ChCl).

### 2.3. Characterization of Materials

#### 2.3.1. XRD Characterization and Testing Conditions

X-ray diffraction (XRD) was used to determine the crystal structures of the different materials. Phase analysis was conducted on the adsorbent samples, and their diffraction patterns were analyzed to determine the peak positions, thereby understanding the composition, internal atomic or molecular structure, and morphology of the samples. The testing conditions for the X-ray diffractometer were as follows: Cu target, Kα radiation (λ = 0.15406 nm), tube voltage and current of 40 kV and 40 mA, respectively, with a scanning speed of 0.5°/min and a 2θ scanning range of 5° to 80° [41].

#### 2.3.2. SEM Characterization and Testing Conditions

Scanning electron microscopy (SEM) was used to characterize the morphology and size of the prepared adsorbents. Owing to the advantage of dispersing particles for clearer SEM characterization results, the particle dispersion process needed to be carried out first. The prepared samples were dissolved in anhydrous ethanol, ultrasonication was started, and the sample was dropped onto a conductive copper plate. Finally, we performed characterization after gold spraying treatment to observe the morphological characteristics of different adsorbents.

#### 2.3.3. N_2_ Adsorption Instrument Characterization and Testing Conditions

The parameter employed for studying the adsorption performance of porous materials is the adsorption/desorption isotherm [42]. For different porous materials, their pore structure and pore size are different, so irreversible adsorption and desorption processes may occur. On the adsorption/desorption isotherm line, it is manifested that the adsorption and adsorption isotherms cannot completely coincide, resulting in hysteresis phenomenon, that is, a hysteresis loop [43]. We can observe the internal pore structure of porous materials from the shape of adsorption/desorption isotherms, and the position and shape of hysteresis loops.

This experiment employed an N_2_ adsorption instrument to measure the adsorption/desorption isotherms of different metal-based adsorbents for N_2_ at 77 K liquid nitrogen temperature. Prior to testing, the samples were subjected to degassing pretreatment at 100 °C for 6 h, from which the specific surface area, pore volume, and pore size of the materials were calculated.

#### 2.3.4. FT-IR Characterization and Testing Conditions

FT-IR can be used for the analysis and identification of substance molecules, as well as for the analysis of molecular structures and chemical bonds. A Fourier transform infrared spectrometer was employed to perform sample infrared analysis. After drying and removing water from the sample, it was mixed with potassium bromide and pressed into tablets. After being secured inside the sample cell, the infrared spectrum was captured. A wavenumber range of 4000 cm^−1^~500 cm^−1^ was observed.

#### 2.3.5. ThermogravimetricCharacterization and Testing Conditions

We performed thermal stability analysis on the material using a thermogravimetric analyzer, recorded the relationship between heat changes and temperature, and increased the temperature to 700 °C at a heating rate of 5 °C/min.

#### 2.3.6. EDS Characterization and Testing Conditions

The incident electron beam was adopted to excite the primary X-rays of the sample material, and the varying energy and wavelengths of the distinctive X-rays were examined to determine the sorts of elements. The composition of each component in the sample material was determined by comparing the content of five elements: C, O, Cr, Cl, and N [13].

### 2.4. Adsorption Experiment

#### 2.4.1. CO_2_ Adsorption Experiment

A BET instrument was used to investigate the samples’ CO_2_ adsorption capability. Prior to the test, for the degassing procedure, the samples were placed into a sample tube at a designated weight (200 mg).

The samples were degassed in N_2_ at 393 K for 6 h to remove moisture and dust, and to desorb impurities such as CO_2_ adsorbed in the sample air, ultimately achieving a pure state. The test results included CO_2_ adsorption isotherms at 298 K and an external pressure range ranging from 0.7~100 kPa. The equation of state was used to fit with the adsorption isotherm.

#### 2.4.2. CO_2_ Adsorption Penetration Experimental Apparatus

**Figure 1:**
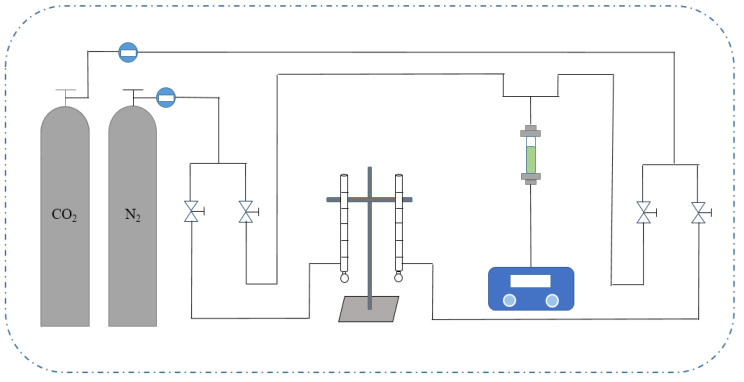
Flow chart of CO_2_ adsorption penetration experiment device.

The experimental setup for MIL-101 adsorption of low concentration CO_2_ is shown in Figure 1. The experimental setup mainly consisted of four systems, namely, a gas supply system, flow detection system, fixed bed adsorption system, and data acquisition system. The gas supply system consisted of a CO_2_ cylinder, an N_2_ cylinder, a pressure gauge, and a pressure reducing valve. N_2_ was used for the purging pretreatment of the experimental device, mixing CO_2_ gas and N_2_ gas. Both gases were controlled by a rotor flowmeter to regulate their flow rate, and then corrected by a soap bubble flowmeter. The fixed bed adsorption system was mainly used for adsorption experiments. The fixed bed size was a high-temperature resistant quartz glass tube with an inner diameter of 5 mm, an outer diameter of 8 mm, and a length of 120 mm. The adsorbent was filled inside the tube. The data acquisition system consisted of a gas sampling port and a CO_2_ gas analyzer, which measured the concentration of CO_2_.

#### 2.4.3. Adsorption Penetration Experiment Process

The specific experimental process for adsorption penetration was as follows:

1. Before starting the adsorption experiment, according to the experimental requirements, 1 g of adsorbent was placed in a quartz glass tube and fixed at both ends with cotton balls.
2. The entire pipeline was blown with N_2_. The adsorption experiment was started once the CO_2_ gas analyzer could not detect any CO_2_ concentration.
3. The two valves of the quartz glass tube were closed, and CO_2_ gas and N_2_ gas were introduced. Preliminary calibration was performed through a glass rotor flowmeter, and a soap bubble flowmeter was leveraged for secondary calibration. The flow ratio of CO_2_ to N_2_ was 15:85; that is, the flow rate of CO_2_ was 6 mL/min, and the flow rate of N_2_ was 34 mL/min.
4. After completing the calibration, the two valves that entered the glass quartz tube were opened, and the two valves that entered the soap bubble flowmeter were closed. The fixed bed adsorption experiment was started and the CO_2_ concentration obtained from the adsorption reaction online was monitored in order to obtain the CO_2_ concentration at different times. When the outlet CO_2_ concentration was detected to be the same as that in the inlet mixed gas, monitoring ceased.
5. The device and the entire gas path were closed, the adsorption experiment was ended, and the adsorption penetration curve was drawn.

#### 2.4.4. Adsorption–Desorption Cycle Experimental Process

To comprehend the stability of adsorbents, it is crucial to research the adsorption desorption cycle. The adsorbent was placed under vacuum conditions at 120 °C for 6 h and the adsorption process was repeated four times. The stability of the adsorbent was determined by comparing its adsorption capacity.

#### 2.4.5. Adsorption Isotherms and Isotherm Equations

The adsorption isotherm equation provides a mathematical description of the adsorption isotherm. The common adsorption isotherm equations include Langmuir equation [44], Freundlich equation [45], Temkin equation [46], Henry equation [47], etc. The Langmuir equation and Freundlich equation are applicable to both physical and chemical adsorption. These two adsorption equations are chosen to characterize the functional relationship in the adsorption isotherm based on the properties of the materials reported in the literature.

(1) Langmuir equation

The Langmuir equation is one of the most famous and widely used adsorption isotherm equations [48], which well describes the adsorption isotherms in the low and medium pressure ranges. When the adsorbate partial pressure in the gas is high and close to the saturated vapor pressure, the equation deviates. This is because the adsorbate at this time can condense in fine capillaries, and the assumption of monolayer adsorption is not valid [49]. The linear expression is as follows:

(1) $$q_{e}=q_{m}\frac{K_{L}p}{1+K_{L}P}$$

*q_e_*: The equilibrium adsorption capacity of the adsorbent,

*q_m_*: The maximum adsorption capacity of the adsorbent,

*p*: CO_2_ partial pressure,

*K_L_*: Langmuir constant.

(2) Freundlich equation

The Freundlich isotherm model is the most widely used model for non-ideal adsorption and multilayer adsorption on heterogeneous surfaces [50]. The Freundlich equation is mainly used for studying the adsorption isotherms of porous solids on adsorbates, which is of great significance for analyzing and predicting adsorption processes. The value of n is related to the surface inhomogeneity of the adsorbent and the energy change in the adsorption process. The larger the value of *n*, the closer the adsorption process is to monolayer adsorption, and the more uniform the adsorbent surface is. On the contrary, the more significant the surface inhomogeneity of the adsorbent, the more obvious the characteristics of multilayer adsorption. Its mathematical expression is:

(2) $$q_{e}=K_{F}P^{\frac{1}{n}}$$

*n*: The degree of heterogeneity in the adsorption process,

*K_F_*: Freundlich constant.

## 3. Results and Discussions

### 3.1. Feature Analysis

#### 3.1.1. XRD Analysis

**Figure 2:**
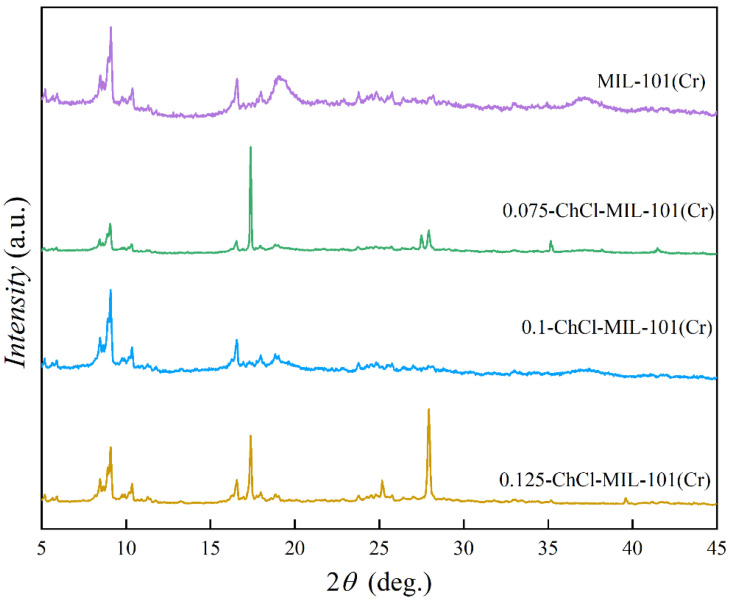
XRD images of MIL-101 (Cr), 0.075-ChCl-MIL-101 (Cr), 0.1-ChCl-MIL-101 (Cr), and 0.125-ChCl-MIL-101 (Cr).

The X-ray diffractometers of adsorbents MIL-101 (Cr), 0.075-ChCl-MIL-101 (Cr), 0.1-ChCl-MIL-101 (Cr), and 0.125-ChCl-MIL-101 (Cr) are shown in the Figure 2. In contrast to MIL-101’s conventional diffraction peaks (Cr), the diffraction peaks of MIL-101 (Cr) at 2θ = 8.4°, 9.04°, and 16.48° confirm the reconstruction of MIL-101 (Cr) [51]. It can be seen that the diffraction peak of MIL-101 (Cr) is relatively sharp and the intensity is high, indicating that the crystal structure of MIL-101 (Cr) is relatively regular. A regular crystal structure usually means that the material has a regular arrangement of pores and adsorption sites, which is conducive to the adsorption of adsorbent molecules. After modification, such as 0.075-ChCl-MIL-101 (Cr), 0.1-ChCl-MIL-101 (Cr), and 0.125-ChCl-MIL-101 (Cr), the shape and intensity of the diffraction peak changed. This indicates that the modification process may change the crystal structure of the material, and then affect the distribution and properties of the adsorption sites. Through comparison of the diffraction peak intensity, it can be found that the modified positions are mainly concentrated at 8.4° and 16.8°.

#### 3.1.2. SEM Analysis

**Figure 3:**
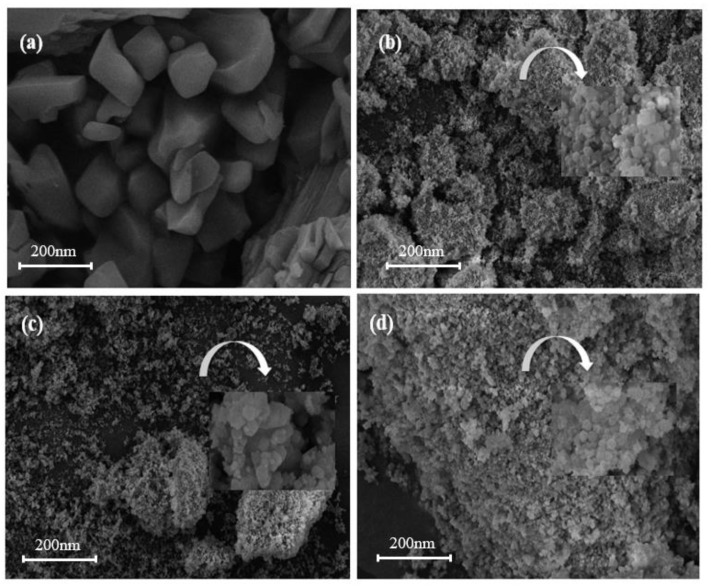
SEM images of (**a**) MIL-101 (Cr), (**b**) 0.075-ChCl-MIL-101 (Cr), (**c**) 0.1-ChCl-MIL-101 (Cr), and (**d**) 0.125-ChCl-MIL-101 (Cr).

**Figure 4:**
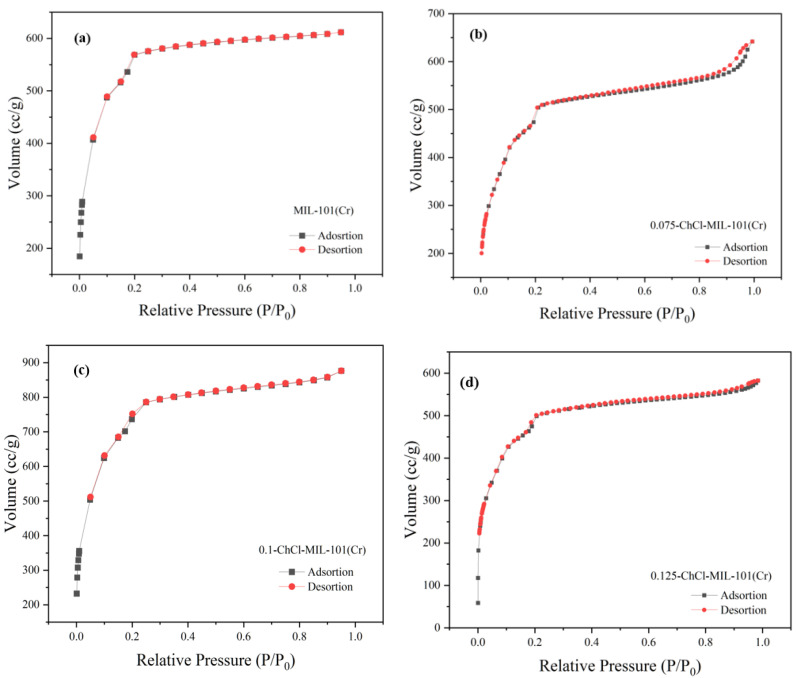
N_2_ adsorption instrument image of (**a**) MIL-101 (Cr), (**b**) 0.075-ChCl-MIL-101 (Cr), (**c**) 0.1-ChCl-MIL-101 (Cr), and (**d**) 0.125-ChCl-MIL-101 (Cr).

The scanning electron microscopy images of adsorbents MIL-101 (Cr), 0.075-ChCl-MIL-101 (Cr), 0.1-ChCl-MIL-101 (Cr), and 0.125-ChCl-MIL-101 (Cr) are shown in the figure. In Figure 3a, The octahedral structure of MIL-101 (Cr) is regular, which is consistent with the findings published in the literature [52]. From Figure 3b, it can be seen that after the combination of the two, the skeleton structure of MIL-101 (Cr) begins to change from smooth to rough because ChCl adheres to the surface of MIL-101 (Cr). In accordance with the findings of the FT-IR investigation, the larger 0.075-ChCl-MIL-101 (Cr) can also be seen for its crystal shape from its interior pores. Furthermore, as ChCl continues to adhere, the uniform channels inside MIL-101 (Cr) begin to fold and bend, which may be due to ChCl filling the MIL-101 (Cr) channels, slightly altering the surface morphology of MIL-101 (Cr). Comparing Figure 4b–d, it can be seen that after ChCl loading, the skeleton structure of MIL-101 (Cr) becomes rougher and more defective, and the internal channel curvature deepens. This suggests that MIL-101 (Cr)’s internal and surface channel structures are affected by ChCl loading. Concurrently, MIL-101 (Cr)’s morphology experiences aggregation and deformation, indicating that ChCl was successfully loaded into MIL-101 (Cr). As the loading amount of ChCl increases, the MIL-101 (Cr) structure becomes rougher and the distortion deepens, indicating that more ChCl molecules are filled in the pores of the MIL-101 (Cr) skeleton.

#### 3.1.3. N_2_ Adsorption Instrument Analysis

The adsorption breakthrough curves of adsorbents MIL-101 (Cr), 0.075-ChCl-MIL-101 (Cr), 0.1-ChCl-MIL-101 (Cr), and 0.125-ChCl-MIL-101 (Cr) are shown in Figure 4. From the isothermal trend lines, MIL-101 (Cr) adsorbents all belong to Class I isotherms, while 0.075-ChCl-MIL-101 (Cr), 0.1-ChCl-MIL-101 (Cr), and 0.125-ChCl-MIL-101 (Cr) belong to Class IV isotherms [53]. The N_2_ adsorption/desorption isotherms of these four different adsorbents appear similar but have significant differences. From Figure 4a, it can be seen that the adsorption–desorption curves almost overlap, indicating that the adsorption/desorption process did not produce a hysteresis loop. This means that the pore structure within the adsorbent is relatively small and contains a large number of microporous structures, with an adsorption capacity of 610 cc/g. From Figure 4b, it can be seen that there is a turning point around P/P_0_ = 0.2, which is the first steep point of the isotherm and represents the saturated adsorption capacity of its single molecule, indicating that its adsorption capacity is complete. However, the adsorption capacity still exceeds that of MIL-101 (Cr) before modification, reaching 641 cc/g. From Figure 4c, it can be seen that although 0.1 mol/L choline chloride modification has the best adsorption and desorption effect on nitrogen gas, reaching 830 cc/g, a narrow hysteresis loop appears around P/P_0_ = 0.18, indicating the presence of a large number of mesopores and relatively few micropores in the 0.1-ChCl-MIL-101 (Cr) sample. From Figure 4d, it is evident that the adsorption–desorption curves are essentially overlapping, with only a narrow hysteresis loop observed at P/P_0_ = 0.17 and P/P_0_ = 0.97, which represents a significant amount of mesopores, while the micropores are comparatively less. The adsorption capacity is 582 cc/g.

**Figure 5:**
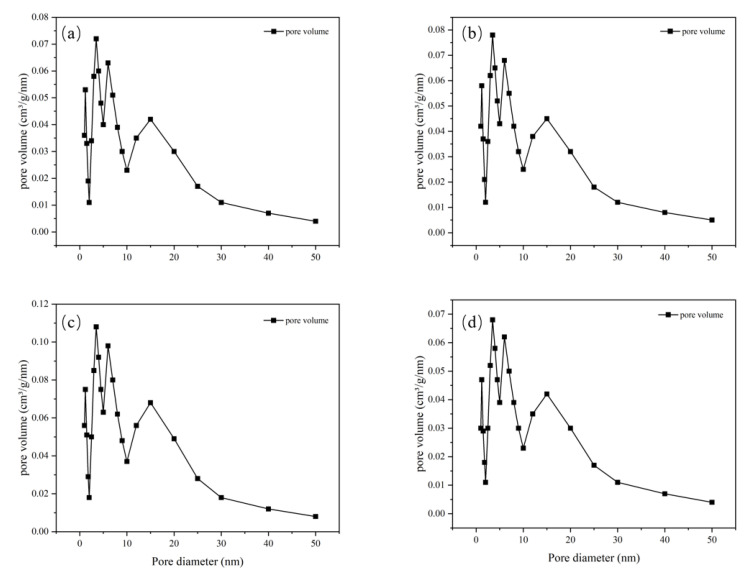
Pore size distribution image for (**a**) MIL-101 (Cr), (**b**) 0.075-ChCl-MIL-101 (Cr), (**c**) 0.1-ChCl-MIL-101 (Cr), and (**d**) 0.125-ChCl-MIL-101 (Cr).

The pore size distribution for MIL-101 (Cr), 0.075-ChCl-MIL-101 (Cr), 0.1-ChCl-MIL-101 (Cr), and 0.125-ChCl-MIL-101 (Cr) is shown in Figure 5. The data analysis of the specific surface area and porosity (BET) of different adsorbents is shown in Table 4. As can be seen from the figure, for MIL-101(Cr), within the pore size range of 0–10 nm, the pore volume fluctuates significantly with multiple peaks, indicating that this material has various pore structures with different pore sizes in the small-pore-size region. As the pore size further increases, the pore volume gradually decreases, and when the pore size is greater than 30 nm, the pore volume approaches zero, suggesting that large-sized pores account for a relatively small proportion in this material. The overall trend of 0.075-ChCl-MIL-101(Cr) is similar to that of MIL-101(Cr), but within the 0–10 nm range, the variation trend and specific values of the pore volume differ from those of MIL-101(Cr). The peak positions and magnitudes are slightly different, which means that after introducing 0.075 mol/L of ChCl, the pore structure of the material has changed to some extent in the small-pore-size region, leading to an adjustment in the pore volume distribution of different pore sizes. For 0.1-ChCl-MIL-101(Cr), within the 0–10 nm pore size range, the variation amplitude of the pore volume is relatively large, and the peaks are distinct.

Compared with the former two, the values of the pore volume at certain pore sizes are different, indicating that as the ChCl content increases to 0.1 mol/L, the influence on the pore structure of the material is further intensified, altering the crystal growth or assembly process of the material and thus affecting the pore size distribution. For 0.125-ChCl-MIL-101(Cr), the pore volume also fluctuates significantly in the 0–10 nm region. However, compared with the other three materials, the details of the pore volume distribution are different. As the ChCl content increases to 0.125 mol/L, the pore structure of the material continues to be modified, influencing the pore formation mechanism of the material at the microscopic level and endowing the pore size distribution with unique characteristics.

#### 3.1.4. FT-IR Analysis

**Figure 6:**
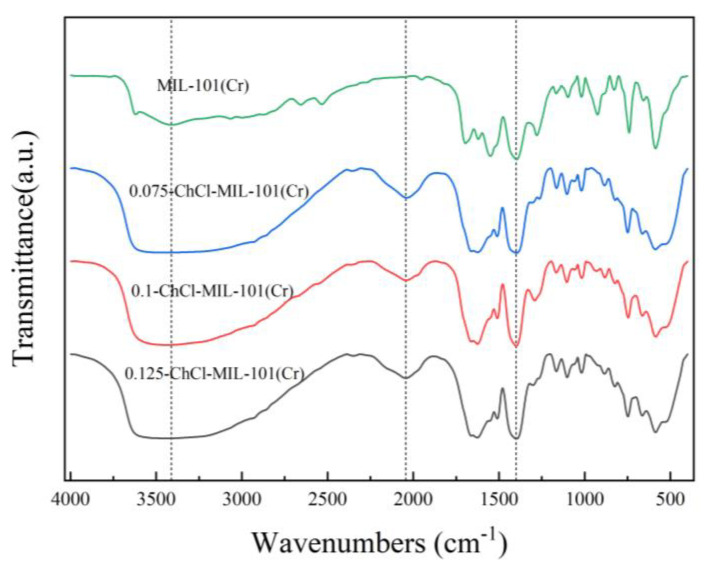
FT-IR images of MIL-101 (Cr), 0.075-ChCl-MIL-101 (Cr), 0.1-ChCl-MIL-101 (Cr), and 0.125-ChCl-MIL-101 (Cr).

The infrared spectra of adsorbents MIL-101 (Cr), 0.075-ChCl-MIL-101 (Cr), 0.1-ChCl-MIL-101 (Cr), and 0.125-ChCl-MIL-101 (Cr) are shown in Figure 6. The Cr-O vibrational absorption peak appeared at 586 cm^−1^, the C-H vibrational peaks adjacent to the benzene ring appeared at 740 cm^−1^ and 827 cm^−1^, an aromatic acid was confirmed at 1279 cm^−1^, the C=O vibrational peak on the symmetrical carboxylate group was located at 1400 cm^−1^, 1549.6 cm^−1^ illustrated to the characteristic peak of the benzene ring, and 3409 cm^−1^ represented the characteristic peak of the carboxylate. This indicates that MIL-101 (Cr) was successfully synthesized, and after adding different concentrations of ChCl, the characteristic peaks at 3000–3400 cm^−1^ showed large pure peaks, indicating the introduction of a large number of hydroxyl groups. This result further proves the successful modification of ChCl.

#### 3.1.5. Thermogravimetric Analysis

**Figure 7:**
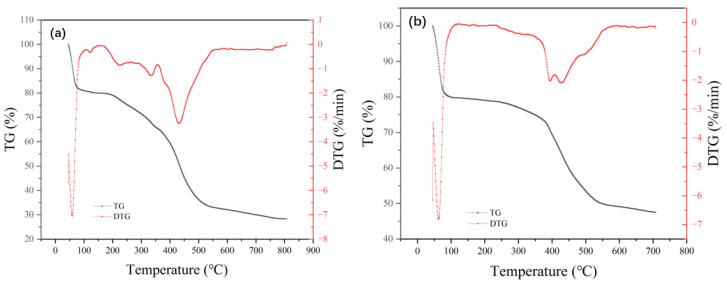

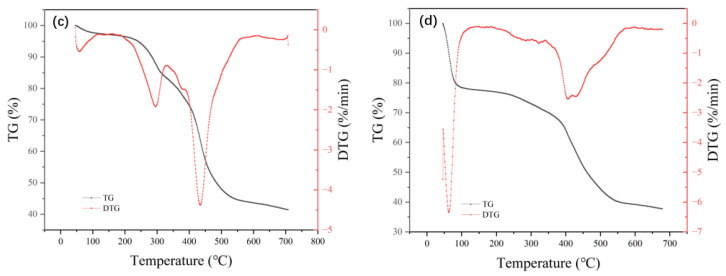
Thermogravimetric images of (**a**) MIL-101 (Cr), (**b**) 0.075-ChCl-MIL-101 (Cr), (**c**) 0.1-ChCl-MIL-101 (Cr), and (**d**) 0.125-ChCl-MIL-101 (Cr).

The thermogravimetric curves of adsorbents MIL-101 (Cr), 0.075-ChCl-MIL-101 (Cr), 0.1-ChCl-MIL-101 (Cr), and 0.125-ChCl-MIL-101 (Cr) are shown in Figure 7. The thermogravimetric analysis of MIL-101 (Cr) mainly consisted of three stages. The escape of water molecules from the larger pore cages (about 34 Å) of the MIL-101 (Cr) structure was the cause of the modest weight loss that occurred before 100 °C. The solvent molecules escaping from the medium pore cage (about 29 Å) caused the weight loss at 100–200 °C; above 200 °C, the MIL-101 (Cr) structure disintegrated and the material lost weight quickly. Owing to the removal of hydroxyl groups from the skeleton, the structure began to collapse [54]. Consequently, MIL-101 (Cr) sustained its structural integrity at an internal temperature of about 200 °C while experiencing a 71.71% mass loss.

**Figure 8:**
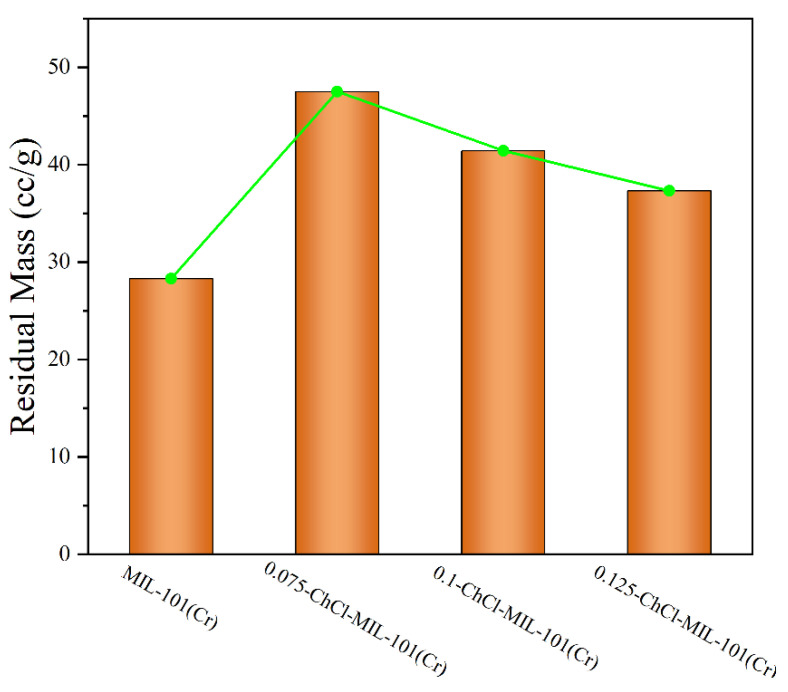
Comparison of residual amounts of each sample.

The comparison of the residual weights of four different materials is shown in Figure 8. After modification with choline chloride, there was a significant enhancement in both thermal stability and residual mass. At temperatures close to 400 °C, the 0.075-ChCl-MIL-101(Cr) can maintain structural integrity, with a mass loss of 52.49%. Compared to the original material, the residual mass increased by 59.54%. However, as the concentration of choline chloride increases, the thermal stability and residual mass start to decline gradually. This is attributed to the decomposition of choline chloride at high temperatures, leading to greater mass loss at higher concentrations, but still surpassing that of the original material.

#### 3.1.6. EDS Analysis

**Figure 9:**
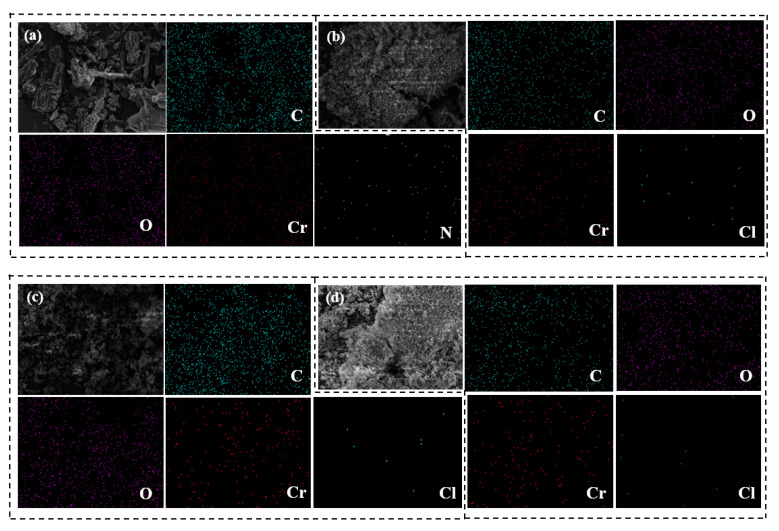
EDS image of (**a**) MIL-101 (Cr), (**b**) 0.075-ChCl-MIL-101 (Cr), (**c**) 0.1-ChCl-MIL-101 (Cr), and (**d**) 0.125-ChCl-MIL-101 (Cr).

The EDS of adsorbents MIL-101 (Cr), 0.075-ChCl-MIL-101 (Cr), 0.1-ChCl-MIL-101 (Cr), and 0.125-ChCl-MIL-101 (Cr) are shown in Figure 9. The data analysis of the elemental composition of different adsorbents is shown in Table 5. During the experiment, some material may be lost from the final sample material after processing. The true composition and proportion of the material can be established by utilizing energy spectrum analysis to examine the kinds and specific gravity of surface distinctive components. The Cr and C elements of a typical MIL-101 (Cr) structure are depicted in Figure 9a. Cr and Cl can be observed in Figure 9b–d, showing a successful composite of the two materials, in line with the findings of the SEM. In addition, based on the content in the table, it can be seen that with an increase in ChCl impregnation amount, the findings of the FT-IR, thermal stability, and SEM analyses are incompatible with the growing quantity of ChCl that is actually loaded into the sample.

### 3.2. Adsorption Experiment

#### 3.2.1. CO_2_ Adsorption Experiment

**Figure 10:**
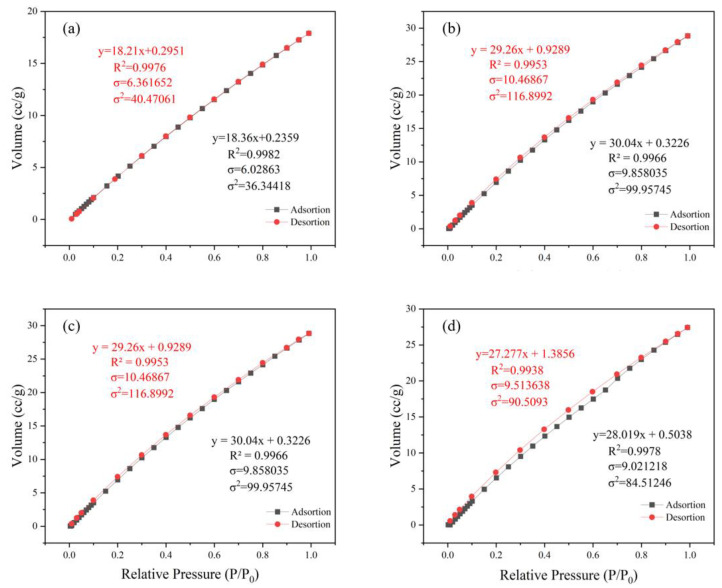
CO_2_ adsorption experiment image for (**a**) MIL-101 (Cr), (**b**) 0.075-ChCl-MIL-101 (Cr), (**c**) 0.1-ChCl-MIL-101 (Cr), and (**d**) 0.125-ChCl-MIL-101 (Cr).

**Figure 11:**
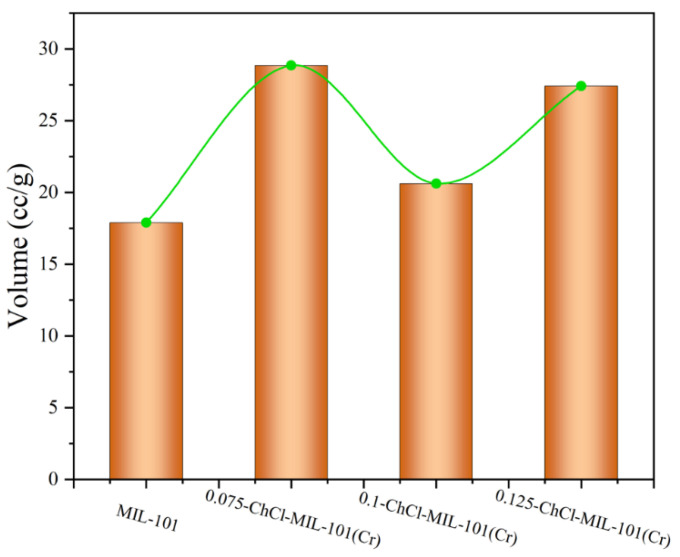
Comparison of CO_2_ adsorption experiment of each sample.

The adsorption properties of n-ChCl-MIL-101 (Cr) and MIL-101 (Cr) modified with different concentrations of choline chloride are shown in the Figure 10. From the figure, it is evident that in the same experimental circumstances, the modification of MIL-101 (Cr) and choline chloride at different concentrations is slightly different. The adsorption capacities of adsorbents MIL-101 (Cr), 0.075-ChCl-MIL-101 (Cr), 0.1-ChCl-MIL-101 (Cr), and 0.125-ChCl-MIL-101 (Cr) are 17.8972 cc/g, 28.8487 cc/g, 20.6239 cc/g, and 27.4263 cc/g, respectively. The comparison of the carbon dioxide adsorption experiments of each sample is shown in Figure 11. The maximum CO_2_ adsorption capacity of choline chloride modified MIL-101 (Cr) at different concentrations is Q_0.075_ > Q_0.125_ > Q_0.1_ > Q_MIL-101 (Cr)_, and the carbon dioxide adsorption capacity increases by 61.191%, 15.235%, 53.243% after modification. It can be inferred that the adsorption property is proportional to the pore size, and it can be inferred that its adsorption performance exhibits a volcano-shaped relationship with the adsorption capacity for carbon dioxide, reaching the maximum adsorption capacity at a concentration of 0.075 mol/L.

#### 3.2.2. Analysis of Adsorption Breakthrough Curve

**Figure 12:**
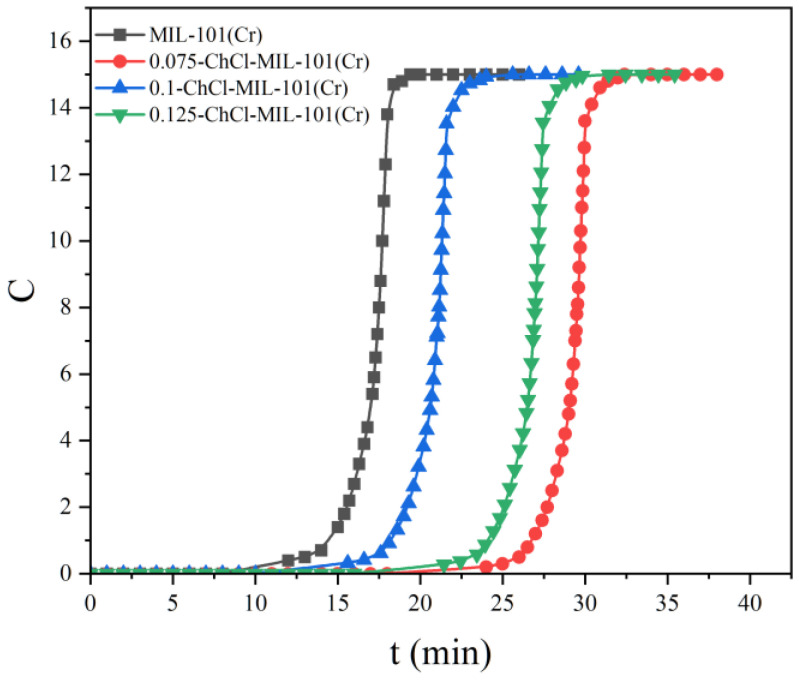
Comparison of CO_2_ adsorption breakthrough curve of each sample.

The adsorption breakthrough curves of adsorbents MIL-101 (Cr), 0.075-ChCl-MIL-101 (Cr), 0.1-ChCl-MIL-101 (Cr), and 0.125-ChCl-MIL-101 (Cr) are shown in Figure 12. From the figure, it is apparent that the CO_2_ adsorption breakthrough curves of the adsorbent materials modified at different concentrations are quite similar, each shifting from a steep to a more gradual slope. The breakthrough time of MIL-101 (Cr) is about 12–17 min, the breakthrough time of 0.075-ChCl-MIL-101 (Cr) is about 24–31 min, the breakthrough time of 0.1-ChCl-MIL-101 (Cr) is about 16–22 min, and the breakthrough time of 0.125-ChCl-MIL-101 (Cr) is about 22–27 min. Among them, the breakthrough time of choline chloride modification with 0.075 mol/L is the longest, indicating that its concentration choline chloride modification has the best adsorption effect on carbon dioxide.

#### 3.2.3. Adsorption Desorption Cycle Experimental Process

**Figure 13:**
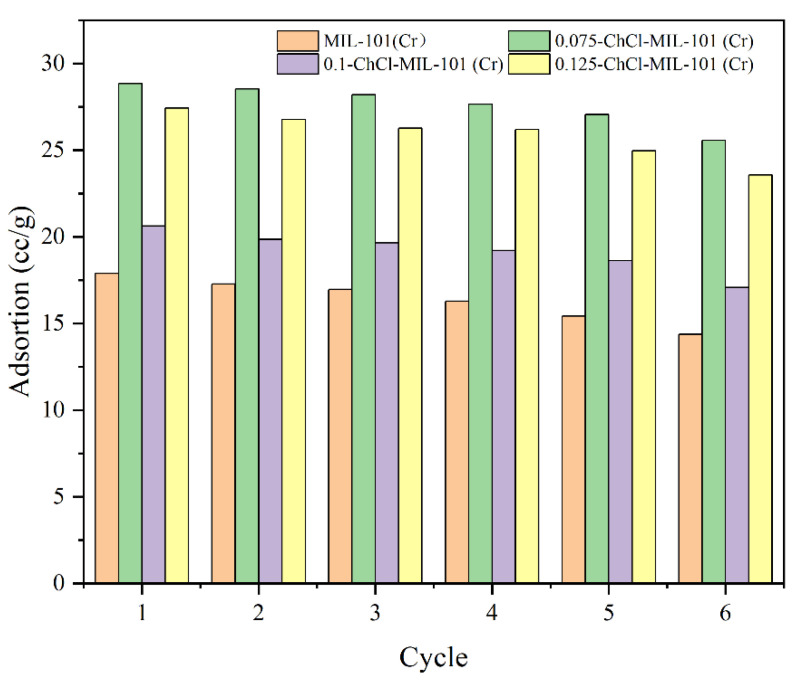
Four cycles of CO_2_ uptake on the samples at 298 K.

The adsorption desorption cycles of the adsorbents MIL-101 (Cr), 0.075-ChCl-MIL-101 (Cr), 0.1-ChCl-MIL-101 (Cr), and 0.125-ChCl-MIL-101 (Cr) are shown in the Figure 13. The data demonstrate no material loss or degradation during the recycling process, with the largest variation in adsorption capacity being less than 4%. This demonstrates the synthetic material’s durability.

#### 3.2.4. Adsorption Isotherms and Isotherm Equations

**Figure 14:**
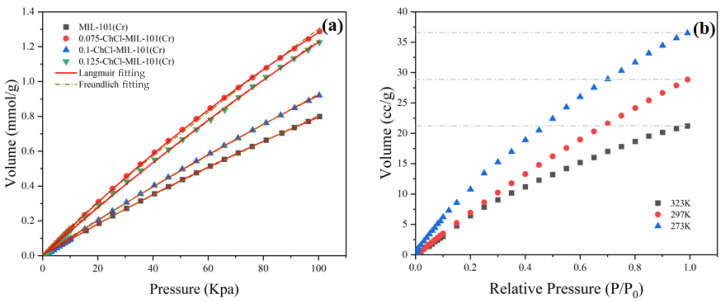
CO_2_ adsorption isotherms: (**a**) Under the environment of 298 K (**b**) 0.075-ChCl-MIL-101 (Cr) under the environments of 273 K, 297 K, and 323 K.

The adsorption of CO_2_ by adsorbent 0.075-ChCl-MIL-101 (Cr) at different temperatures is shown in Figure 14. The findings show that the temperature has an inverse relationship with the equilibrium amount of carbon dioxide adsorption. This is because exothermic adsorption is the norm [55]. Therefore, raising the temperature will decrease adsorption as long as the adsorption equilibrium is reached. It was discovered that both the Langmuir and Freundlich fitting results of CO_2_ isotherms may accurately fit the isotherm data. However, it was also discovered that the Freundlich fitting effect outperforms Langmuir for materials treated with choline chloride, suggesting that the current situation cannot be adequately described by the assumption of monolayer adsorption. In multilayer adsorption, the first layer of adsorbent molecules binds to the active sites on the surface of the adsorbent, and the adsorption energy of these active sites is different due to the surface heterogeneity. As the adsorption proceeds, the molecules of the subsequent adsorption layer interact not only with the adsorbent surface, but also with the adsorbed molecules. Adsorbents have the capability to adsorb gas molecules in layers. This suggests that surface binding in adsorption behavior is not uniform. Stated differently, there is an unequal distribution of adsorption energy on the material’s surface. This suggests that the composite material was effectively loaded with choline chloride, hence augmenting the chemical adsorption force within the material.

The isothermal constants determined using nonlinear fitting of equilibrium data are summarized in Table 6. The temperature and characteristics of the adsorbent and adsorbate are connected to the Langmuir equilibrium constant (KL), whose value increases with the adsorbent’s adsorption performance [56]. After loading choline chloride, the chemical composition of KL increased, indicating that its adsorption performance was improved, and its value reflects the difficulty of adsorption behavior [57]. Based on the actual experimental data, the highest adsorption capacity among different choline chloride modifications is 0.075-ChCl-MIL-101 (Cr).

#### 3.2.5. Analysis of CO_2_/N_2_ Adsorption Selectivity

**Figure 15:**
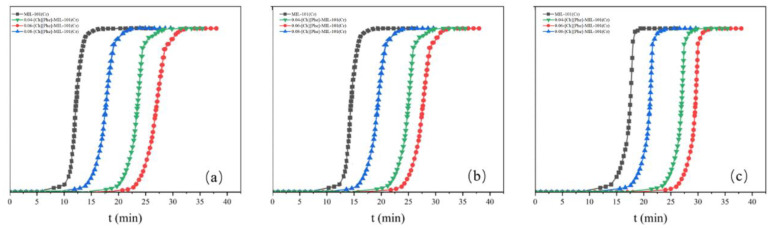
Adsorption breakthrough curves at different concentrations: (**a**) CO_2_:N_2_ = 0.12:0.88 (**b**) CO_2_:N_2_ = 0.09:0.91 (**c**) CO_2_:N_2_ = 0.06:0.94.

The CO_2_/N_2_ Adsorption Selectivity of the adsorbents MIL-101 (Cr), 0.075-ChCl-MIL-101 (Cr), 0.1-ChCl-MIL-101 (Cr), and 0.125-ChCl-MIL-101 (Cr) are shown in the Figure 15. From the perspective of adsorption kinetics, as the concentration of carbon dioxide in the mixed gas decreases, the adsorption breakthrough times of the four adsorption materials all show a gradually increasing trend. Specifically, the adsorption breakthrough time of 0.04-[Ch][Phe]-MIL-101(Cr) is extended by approximately 4 min, that of 0.06-[Ch][Phe]-MIL-101(Cr) is extended by about 5 min, and that of 0.08-[Ch][Phe]-MIL-101(Cr) is extended by around 6 min. This phenomenon indicates that a lower carbon dioxide concentration slows down the diffusion rate of gas molecules within the pore channels of the adsorbent and reduces the effective collision frequency with the adsorption active sites, thus delaying the process of adsorption saturation and leading to an increase in the adsorption breakthrough time.

Moreover, although the adsorption breakthrough times of the four materials all increase with the decrease in concentration, the increment gradually decreases. This shows that under low-concentration conditions, the adsorption selectivity of the adsorbent for carbon dioxide does not change in a simple linear manner, but there is a certain concentration threshold effect. When the carbon dioxide concentration decreases to a certain extent, the ability of the adsorbent to recognize and selectively adsorb carbon dioxide molecules can still be maintained at a relatively high level, causing the increase in the adsorption breakthrough time to slow down. This characteristic proves that these four materials still possess high CO_2_/N_2_ adsorption selectivity in an environment with a low carbon dioxide concentration. The underlying mechanism may stem from the special pore-channel structure and surface chemical properties of the adsorbent, which can preferentially recognize and adsorb carbon dioxide molecules, enabling effective separation of carbon dioxide from nitrogen even in a complex mixed-gas system with a low carbon dioxide content.

### 3.3. Analysis of the Mechanism of MIL-101 (Cr) Modified by Choline Chloride

The modification of MIL-101(Cr) with choline chloride enhances the carbon dioxide adsorption performance. The mechanism mainly involves aspects such as changes in the microstructure, alterations in the surface properties, and enhancement of the chemical interactions.

#### 3.3.1. The Change in Microstructure Promotes Adsorption

During the modification process, choline chloride interacts with the MIL-101 (Cr) framework through weak interactions such as hydrogen bonds and van der Waals forces, inducing the rearrangement of the framework structure and increasing the pore volume. This reduces the mass transfer resistance of carbon dioxide molecules within the material, allowing them to diffuse more rapidly to the adsorption active sites and accelerating the adsorption kinetic process. For example, in experiments, the change in the adsorption breakthrough time of the modified material reflects this advantage. The breakthrough time of 0.075-ChCl-MIL-101 (Cr) is prolonged, indicating that it can adsorb more carbon dioxide under the same conditions.

#### 3.3.2. Generation of Active Sites

Some functional groups in choline chloride, such as hydroxyl (-OH) and amino (-NH_2_) groups, will interact with the metal sites or organic ligands in MIL-101 (Cr), generating new active sites on the material surface or within the pores. These active sites can undergo chemical reactions with carbon dioxide molecules to form chemical bonds or chemisorption complexes. Hydroxyl can react with carbon dioxide through an acid–base neutralization reaction to form bicarbonate ions, etc., thus achieving the chemisorption of carbon dioxide.

#### 3.3.3. Change in Electron Cloud Density

The presence of choline chloride can alter the electron cloud density around the metal ions in MIL-101 (Cr). For the Cr ions in MIL-101 (Cr), the change in electron cloud density affects their coordination ability with carbon dioxide molecules. The oxygen atoms in carbon dioxide molecules have lone pairs of electrons and can form coordination bonds with metal ions. After modification with choline chloride, the change in the electron cloud density around Cr ions enhances their coordination with carbon dioxide molecules, making it easier for carbon dioxide to be adsorbed and stabilized on the surface of the adsorbent.

## 4. Conclusions

This study employs the hydrothermal method to prepare MIL-101 (Cr), modifies the composite material using a one-step process, and effectively packs various choline chloride concentrations to produce a novel kind of carbon capture material. Choline chloride is added to the composite material, increasing its pore volume and decreasing its mass transfer resistance, accelerating the adsorption of CO_2_. MIL-101 has a more advantageous adsorption behavior due to its unique pore cage structure. A specific concentration of choline chloride added to composite materials allows them to keep their crystal structure. However, as the load increases, molecules fill the pores, reducing the material’s specific surface area and pore size to differing degrees.

The CO_2_ adsorption isotherm of the loaded material is more closely fitted by the Freundlich equation than by the Langmuir equation, suggesting that the adsorption behavior of materials treated with choline chloride on irregular surfaces is more consistent with multi-layer non-ideal adsorption behavior. The introduction of choline chloride enhances the sample’s adsorption capacity for CO_2_. The most effective adsorption effect on CO_2_ is shown by the composite material at a concentration of 0.075 mol/L. Under the conditions of 293 K and 100 kPa, a saturation adsorption capacity of 28.8487 cc/g is achieved, turning it into a carbon dioxide adsorbent with a wide range of potential uses. Since adsorption is an exothermic process, an increase in temperature causes a decrease in the adsorption capacity of the material. In conclusion, this study offers us a fresh approach to combination strategies.

The research findings of this study have significant practical implications at present, providing a new material option for the optimization of carbon capture technologies in the industrial field. In existing low-concentration carbon dioxide capture scenarios, such as emission sources in thermal power plants and cement plants, traditional adsorption materials suffer from problems such as insufficient adsorption capacity and poor selectivity.

In terms of future research directions, this study has opened up several important paths for follow-up exploration. First, it is possible to further investigate the synergistic mechanisms between different modifiers and MIL-101(Cr). Instead of being limited to choline chloride, other organic or inorganic modifiers with special functions can be introduced, and more diversified composite methods can be explored to achieve further breakthroughs in the material’s adsorption performance, for example, enhancing its resistance to specific gas impurities and improving its adsorption stability under complex working conditions. Second, this study has only examined a limited range of experimental conditions. In the future, the experimental scope can be expanded to study the adsorption performance of the material under a wider range of temperatures, pressures, and gas compositions, and a more complete performance database can be established to provide more comprehensive data support for practical engineering applications.

## Figures and Tables

**Table 1:** Comparison of CO_2_ adsorption capacity of different MIL materials.

MOF Materials	Specific Surface Area (m^2^/g)	Average Pore Diameter (nm)	Pore Volume (cm^3^/g)	CO_2_ Adsorption Capacity (mg/g)	Reference
MIL-101 (Cr)	2546.69	0.98	1.95	44.21	[26]
MIL-101 (Fe)	1129.43	3.76	0.93	36.28	[26]
MIL-101 (Al)	1847.57	1.85	1.43	32.41	[26]
MIL-100 (Fe)	1501	1.33–2.7	1.01	63.27	[27]
Ethylenediamine modified MIL-101	2215	1.24	1.69	107.35	[28]
Modified MIL-101 pentaethylenehexamine	1791	2.04	1.37	58.94	[29]
MIL-53	2579	0.57	0.72	106.72	[30]
NH_3_-MIL-53	-	-	0.81	132.58	[30]

**Table 2:** Summary of materials.

Material	Chemical Formula	Purity	Manufacturer
Chromium nitrate nonahydrate	Cr(NO_3_)_3_·9H_2_O	99%	Macklin (Shanghai, China)
Terephthalic acid	HOOCC_6_H_4_COOH	99%	Macklin (Shanghai, China)
N,N-Dimethylformamide (DMF)	HCON(CH_3_)_2_	99%	Macklin (Shanghai, China)
Anhydrous ethanol	C_2_H_5_OH	AR	Macklin (Shanghai, China)
Hydrofluoric acid	HF	AR	Macklin (Shanghai, China)
Deionized water	H_2_O	--	Self-prepared
Carbon dioxide	CO_2_	99.99%	Huayang Gas (Foshan, China)

**Table 3:** Summary of experimental equipment.

Experimental Equipment	Model	Manufacturer
Electronic Balance	F2004N	Shanghai Scientific Precision Instrument Co., Ltd. (Shanghai, China)
Magnetic Stirrer	DF-101S	Henan Yuhua Instrument Co., Ltd. (Zhengzhou, China)
Electrothermal Constant-Temperature Forced-Air Drying Oven	OHG-905385	Shanghai Xinmiao Medical Instrument Manufacturing Co., Ltd. (Shanghai, China)
High-speed Centrifuge	TD4K-Z	Changsha Dongwang Experimental Instrument Co., Ltd. (Changsha, China)
Hydrothermal Reaction Kettle	YZHR-100	Beijing Yanzheng Biotechnology Co., Ltd. (Beijing, China)
Recirculating Water-type Multi-purpose Vacuum Pump	SHZ-D-III	Henan Yuhua Instrument Co., Ltd. (Zhengzhou, China)
Micropipette (20–200 μL)	20–200 μL	Dilong Xunchuang Experimental Instrument Co., Ltd. (Beijing, China)
Numerical Control Ultrasonic Cleaner	KQ5200DE	Kunshan Ultrasonic Instrument Co., Ltd. (Suzhou, China)
Scanning Electron Microscope	Quanta 200F	FEI Company (Eindhoven, The Netherlands)
Nitrogen Adsorption-Desorption Analyzer	ASAP2020	Micromeritics Instrument Corporation (Norcross, GA, USA)
X-ray Powder Diffractometer	EMPYREAN	PANalytical B.V. (Almelo, The Netherlands)
Thermogravimetric Analyzer	CW-TE240	Shanghai Chengwei Instrument Technology Co., Ltd. (Shanghai, China)
Fourier Transform Infrared Spectrometer	VERTEX 70	Bruker Corporation (Ettlingen, Germany)
Carbon Dioxide Adsorption Analyzer	LYT-1971-2011	Shandong Shengtai Instrument Co., Ltd. (Jinan, China)

**Table 4:** BET data analysis of different adsorbents.

MOF Materials	Specific Surface Area (m^2^/g)	Pore Volume (cm^3^/g)	Average Pore Diameter (nm)
MIL-101	2088.05	0.99	1.89
0.075-ChCl-MIL-101(Cr)	1817.02	0.99	2.18
0.1-ChCl-MIL-101(Cr)	1384.47	0.72	1.95
0.125-ChCl-MIL-101(Cr)	1777.81	0.90	2.02

**Table 5:** Elemental composition data analysis of different adsorbents.

	MIL-101(Cr)		0.075-ChCl-MIL-101(Cr)		0.1-ChCl-MIL-101(Cr)		0.125-ChCl-MIL-101(Cr)	
Elemental	Wt%	At%	Wt%	At%	Wt%	At%	Wt%	At%
C	44.47	59.51	52.32	68.79	59.04	73.14	55.73	68.60
O	37.06	37.24	30.62	24.03	25.43	19.06	28.13	21.63
Cr	18.47	3.25	17.51	2.94	11.24	1.25	10.21	1.19
Cl	0	0	3.16	4.24	4.29	6.55	5.93	8.58

**Table 6:** Langmuir and Freundlich constants of CO_2_ adsorption on samples at 298 K.

	Langmuir			Freundlich		
	q_m_ (mmol·g^−1^)	K_L_ (Kpa/mmol)	R^2^	q_m_ (mmol·g^−1^)	n	R^2^
MIL-101(Cr)	5.19465	0.00181	0.999	0.01177	1.0902	0.999
0.075-ChCl-MIL-101(Cr)	6.37377	0.00252	0.999	0.02144	1.1177	0.999
0.1-ChCl-MIL-101(Cr)	7.94415	0.00191	0.999	0.01254	1.07	0.999
0.125-ChCl-MIL-101(Cr)	8.94544	0.00239	0.999	0.01802	1.1189	0.999

## Data Availability

The original contributions presented in this study are included in the article. Further inquiries can be directed to the corresponding author.
